# Gitelman Syndrome: A Rare Cause of Seizure Disorder and a Systematic Review

**DOI:** 10.1155/2019/4204907

**Published:** 2019-02-05

**Authors:** Muhammad Asim Shahzad, Maryam Mukhtar, Asrar Ahmed, Waqas Ullah, Rehan Saeed, Mohsin Hamid

**Affiliations:** ^1^Resident Physician, Louis Weiss Memorial Hospital, Chicago, IL, USA; ^2^Independent Research Scholar, Fauji Foundation Hospital, Rawalpindi, Pakistan; ^3^Resident Physician, Abington Hospital-Jefferson Health, Abington, PA, USA

## Abstract

Gitelman syndrome is one of the few inherited causes of metabolic alkalosis due to salt losing tubulopathy. It is caused by tubular defects at the level of distal convoluted tubules, mimicking a thiazide-like tumor. It usually presents in late childhood or in teenage as nonspecific weakness, fatigability, polyuria, and polydipsia but very rarely with seizures. It is classically associated with hypokalemia, hypomagnesemia, hypocalciuria, hyperreninemia, and hyperaldosteronism. However, less frequently, it can present with normal magnesium levels. It is even rarer to find normomagnesemic patients of GS who develop seizures as the main complication since hypomagnesemia is considered the principal etiology of abnormal foci of seizure-related brain activity in GS cases. Interestingly, patients with GS are oftentimes diagnosed during pregnancy when the classic electrolyte pattern consistent with GS is noticed. Our case presents GS with normal serum magnesium in a patient, with seizures being the main clinical presentation. We also did a comprehensive literature review of 122 reported cases to show the prevalence of normal magnesium in GS cases and an overview of clinical and biochemical variability in GS. We suggest that further studies and in-depth analysis are required to understand the pathophysiology of seizures in GS patients with both normal and low magnesium levels.

## 1. Materials and Methods

Two different databases (PubMed and Scopus) were searched for all case reports and review articles previously published on GS syndrome. Moreover, after taking informed consent from our patient, we included data from the electronic medical record system to use this information for publication purposes.

## 2. Case Presentation

A 22-year-old female was brought to the hospital with the complaint of vomiting, generalized weakness, and two episodes of witnessed generalized tonic-clonic seizures 24 hours prior to the time of admission. She had about 5 episodes of nonbloody nonbilious vomiting. She was nonverbal at baseline but was reported to be more lethargic than usual and had a poor oral intake for the last 2 days and appeared to be in pain. Review of the system was negative for any previous episodes of seizures in the past, fever, diarrhea, abdominal pain, history of diuretic or laxative abuse, any periorbital puffiness, and extremities swelling. She was given lorazepam followed by successful resolution of seizures.

On physical examination, she was having borderline low blood pressure close to her baseline (105/56) with HR of 80, RR 18, O_2_ sat. 100% on room air. Systemic examination was otherwise unremarkable without any overt signs of dehydration.

EKG showed U waves and nonspecific T wave changes. Pertinent labs showed serum blood urea nitrogen (BUN) and creatinine (Cr) of 16 and 0.77, respectively. Serum electrolytes showed serum sodium (Na) of 150 mEq/L, serum potassium (K) of 1.4 mEq/L, serum magnesium (Mg) of 2.8 mg/dL, and serum bicarbonate (HCO_3_) of 35 mEq/L. Urine electrolytes included urine K 22 mEq/L, urine Na 121 mEq/L, and urine Cl 146 mEq/L. Her transtubular potassium gradient (TTKG) was 6.82. Complete blood count and liver function panel were within normal limits. Plasma renin activity (PRA) was 0.33 ng/ml/hr, serum aldosterone/K ratio of 1/1.4, and aldosterone/plasma renin ratio of 3. Differential included primary hyperaldosteronism, vomiting, and Bartter/Gitelman syndrome.

EEG showed abnormal epileptiform activity in the brain consistent with seizure. Low normal BP, high urine Cl with urine Ca, and history negative for laxative/diuretic intake made GS the more likely differential. Later on, biallelic identification of inactivating SLC12A3 mutation confirmed the diagnosis of GS.

Patient's condition improved with aggressive K replenishment and antiepileptics in the medical ICU. She was later discharged in a medically stable condition and advised to follow-up with nephrologist and neurologist as an outpatient.

## 3. Literature Search

The available literature was systematically searched by three authors independently to retrieve all available material on variable clinical and metabolic presentations in Gitelman syndrome. There was no language filter placed, and articles were collected from their inception till May 2018, using the MEDLINE, Cochrane, Embase, and Scopus databases. Different MeSH terminologies such as “Gitelman,” “Gitelman syndrome,” “Gitelman disease,” and “GS” were combined using the Boolean operators “AND” and “OR” with the terms “hypomagnesemia,” “low magnesium,” “serum magnesium,” “plasma magnesium,” and “magnesium levels.” Another author collected few articles through manual search using the reference list of all retrieved publications through the aforementioned search strategy.

## 4. Results and Statistical Analysis

### 4.1. Literature Retrieval and the Results

After a thorough computer literature search, careful verification of references, and screening based upon the titles and abstracts, 122 cases of GS patients from 100 articles were identified for selection [1–100]. It was ensured that repetitive cases in these articles were excluded. Out of these 100 articles, data were also extracted from articles published in languages other than English.

### 4.2. Patients Description

There were a total of 122 patients including 45% (*n*=55) males and 65% (*n*=77) females. The age of female patients ranged from 4.8 months to 79 years (mean age 28.5 years), whereas for males, it ranged from 7 months to 80 years (mean age of 27.8 years). The description of patients included in this study is listed in Table 1.

### 4.3. Spectrum of Clinical Presentation and Associations

Clinical presentation of Gitelman syndrome was found to be highly variable in the reported patient population. About 30% (*n*=36/122) of the patients, including 14% (*n*=17/122) pregnant patients, were having nonspecific muscle cramps, weakness, fatigability, and anorexia, as the main presentation. These were likely due in part to hypokalemia and hypomagnesemia. About 12% (*n*=15/122) of the patients had extremities weakness out of which 7% (*n*=9/122) presented with bilateral lower limb weakness/paralysis and the rest of them had quadriplegia as initial presentation. Interestingly, 10.6% (*n*=12/122) of patients had perioral numbness and symptoms related to tetany/carpopedal spasm as first signs of Gitelman. About 6% of patients had polydipsia, polyuria/enuresis, and salt craving as presenting complaint; however, almost half of the total reported patients had some degree of polydipsia and polyuria in addition to main presenting clinical symptoms. Seven percent (*n*=9/122) of patients were completely asymptomatic and were diagnosed with routine lab work, either during routine clinical visits or perioperatively. Only 5.7% of patients (*n*=7/122) had GI-related issues such as anorexia, vomiting, constipation, abdominal pain, and weight loss as the main complaint. About 7 cases had no mention of the presenting complaints. Rest of the patients had their own unique features as seen in Table 1. Our patient had a unique presentation of generalized tonic-clonic seizure despite normal serum Mg levels, which has not been previously reported in the literature. GS was found to be most commonly associated with pseudogout and CPPD crystal deposition in about 10% of patients. Other associations included but not limited to Sjogren's syndrome in 4%, chondrocalcinosis in 3%, and diabetes mellitus (both type 1 and type 2) and primary hyperaldosteronism in about 2% each. A less common association is seen with empty sella syndrome in 2 patients. Seizure disorder as a possible association with GS was previously reported in only one case by Beltagi et al., most likely due to hypomagnesemia [15]. Our patient, however, was unique with no prior history of epilepsy and had a seizure as the very first presentation with normal magnesium levels.

### 4.4. Complications Related to Gitelman Syndrome

Complications related to renal, cardiac, and endocrine systems have frequently been reported in the previous cases. Cardiac manifestations ranged from electrolytes related, asymptomatic ECG changes including prolonged Qtc, nonspecific T and U waves to pericardial effusion, and ventricular fibrillation. Reported renal pathologies included glomerulonephritides such as MPGN, FSGS, membranous nephropathy, and also cases of tubulointerstitial nephritis and renal tubular acidosis (RTA). Thyrotoxic periodic paralysis and hypokalemic periodic paralysis were also seen in a few cases. However, it must be noted that it is rare for two different renal entities to occur at the same time, and several of the studies did not confirm the diagnosis of GS by identifying the inactivation gene mutation leaving open the possibility that underlying pathology may not have been actually Gitelman's. Long-term follow-up is usually required to observe for these complications; our patient, however, had no further follow-up in our hospital and was referred to the neurologist care.

### 4.5. Diagnosis and Management with Outcomes

Except for one case (*n*=1/122), where there is no mention of the diagnostic method, genetic testing was utilized in 42% (*n*=52/122) cases, to definitively diagnose GS. The specific mutations to help make the diagnosis can be seen in Table 1. Almost 56% of patients (*n*=68/122) were diagnosed based on the presenting electrolytes abnormalities including serum and urine Na, K, Mg, and Ca used adjunctively with PAR concentration. Although the supportive testing with electrolytes and supplementary tests were highly suggestive of GS in these 68 cases, genetic tests were not done for various reasons. These included lack of resources, nonavailability of genetic test, and loss of follow-up by the patients to be the major ones.

Of note is the serum Mg level in the reported cases. Considering the normal range to be between 0.7 and 1 mmol/L (1.5–2 mEq/L; 1.7–2.4 mg/dL), 55% (*n*=66/122) patients had hypomagnesemia, i.e., <0.7 mmol/L, whereas 20% (*n*=25/122) had levels 0.7 mmol/L and above. In 31 cases, serum magnesium levels were not reported. These levels were important as the clinical severity of presentation was reflected by the degree of hypomagnesemia.

Electrolytes replacement, NSAIDs, and potassium-sparing diuretics with and without ACE In/ARB's were the mainstay of treatment in almost all of the cases. Outcomes and prognosis were remarkable, and patients fully recovered from their acute presenting symptoms with exception of a few cases. These few cases reported persistent electrolytes abnormalities such as hypokalemia, metabolic alkalosis, hypomagnesemia, occasional paralysis and neurological symptoms, and treatment-related complications (indomethacin-related GI upset and bleeding). Recovery in the other cases is being defined as a sustained increase in electrolytes with magnesium >2, potassium >4, and significant improvement in the symptoms. Around 22% (*n*=28/122) cases did not comment on the outcomes.

## 5. Discussion

However, GS can also present with normal serum magnesium levels, and in one case, it has been reported to be in around 20–40% of GS cases [101]. From our review of around 122 cases, 20% (*n*=25/122) patients had serum magnesium levels >0.7 mmol/L. Both the groups of GS patients with normal and low magnesium levels largely stay asymptomatic and present later in life. Most present in teenage or adulthood with nonspecific generalized weakness or muscle cramps/fatigability, polyuria, and polydipsia [103]. However, seizure disorder has very rarely been reported as one of the main presenting complaints. Hypomagnesemia and metabolic alkalosis have been proposed as the pathophysiological basis of these rarely reported seizure disorders. Our case reports are unique in this sense that the patient of GS presented with seizure despite having normal serum magnesium levels. In our literature review, only one patient who was reported by Beltagi et al. [15] presented with somnolence and altered mental status and had a focal seizure as a complication. Even in that case, hypomagnesemia can be considered as the cause of epileptiform activity on EEG. This observation prompts us to consider causes other than hypomagnesemia as a culprit of seizure disorder, whenever evaluating the patient with GS. The final diagnosis of GS is based on the triad of clinical symptoms, biochemical abnormalities, and genetic testing [103]. Genetic testing is recommended for all patients, and the diagnosis is confirmed with the biallelic identification of inactivating SLC12A3 mutations [104]. We emphasize after this literature review that contrary to common clinical practice, overall clinical picture with more emphasis on genetic testing is a better strategy to clinch the diagnosis, and the diagnosis of GS can still be made even with normal serum magnesium levels.

### 5.1. Treatment

Most patients with GS remain untreated. The observation that chondrocalcinosis is due to magnesium deficiency argues clearly in favor of magnesium supplementation [15]. Most asymptomatic patients with GS remain untreated and undergo ambulatory monitoring, once a year, generally by nephrologists. Lifelong supplementation of magnesium and potassium is mandatory [105]. Cardiac workup should be performed to screen for risk factors of cardiac arrhythmias. All GS patients are encouraged to maintain a high-sodium diet. In general, the long-term prognosis of GS is excellent. Health education with annual regular nephrologist follow-up to evaluate for any developing complications seems to be a reasonable approach. As mentioned in the abstract, GS can be first identified during pregnancy when classic electrolyte abnormalities are noticed on the lab work [106]. Successful pregnancy is possible in majority of the patients; however, miscarriages have also been reported in the literature, which alludes to regular nephrologist follow-up during pregnancy.

## 6. Conclusion

- GS with variable biochemical presentation, i.e., normal serum magnesium level is a rare but potentially possible finding seen in various clinical settings
- Although exceedingly rare, seizure disorder can be the main clinical presentation of GS
- Causes other than low magnesium levels should be sought for the explanation of seizure disorder in GS
- Further studies are recommended to better understand the pathophysiology of abnormal epileptiform activity in GS
- Successful pregnancy is possible in majority of the patients; however, miscarriages have also been reported in the literature, which alludes to regular nephrologist follow-up in the pregnant GS patient

## Figures and Tables

**Table 1:** Summary of the literature review.

Demographics	Total (%)	Associations	Total (%)
Age, mean	31	Pregnancy	17 (14)
Range	0.3–80 years	Calcium pyrophosphate deposition disease (CPPD)	7 (5.7)
Males	45 (36)	Sjogren syndrome	5 (4)
Females	77 (63)	Chondrocalcinosis	4 (3.3)
*Presentation*		Thyrotoxicosis hypokalemic periodic paralysis (THPP)	2 (1.6)
Weakness	52 (43)	Empty sella syndrome	2 (1.6)
Cramps	23 (19)	Type 2 diabetes	2 (1.6)
Carpopedal spasms	11 (9)	Primary aldosteronism	2 (1.6)
Nausea, vomiting	7 (6)	Type 1 diabetes	1 (0.8)
Nocturia	6 (5)	Pemphigus vegetans	1 (0.8)
Paralysis	5 (4)	Mitochondrial encephalopathy	1 (0.8)
Numbness	5 (4)	Varicose veins	1 (0.8)
Joint pain, arthritis	4 (3) each	Fanconi syndrome	1 (0.8)
Muscle pain	3 (2)	Autosomal dominant familial neurohypophyseal diabetes insipidus	1 (0.8)
Polydipsia	3 (2)	Syndrome of inappropriate ADH secretion	1 (0.8)
Sicca symptoms	3 (2)	Familial Mediterranean fever	1 (0.8)
Hypokalemic paralysis	2 (1.6)	Parathyroid adenoma	1 (0.8)
Syncope	2 (1.6)	Pancreatic cancer	1 (0.8)
Salt craving	2 (1.6)	Hashimoto thyroiditis	1 (0.8)
Thirst, palpitations, frequent micturition, somnolence	1 (0.8) each	Scleroderma	1 (0.8)
Nausea, vomiting	7 (6)	Crowded lens syndrome	1 (0.8)
Paralysis	5 (4)	Transient hypophosphatemia of infancy	1 (0.8)
Visual abnormalities	4 (3)	Pseudotumor cerebri	1 (0.8)
Failure to thrive	4 (3)	Gout	1 (0.8)
Loss of appetite	4 (3)	Graves disease	1 (0.8)
Respiratory distress	3 (2)	*Serum Electrolytes*	*Total (%)*
Arthralgias	2 (1.6)	*Sodium*	
Headache	2 (1.6)	Normal range (135–145 mEq/L)	46 (38)
Diarrhea	2 (1.6)	Low	15 (12)
Raynaud's phenomenon	2 (1.6)	*Potassium*	
Incontinence	1 (0.8)	Normal range (3.5–5 mEq/L)	14 (11)
Insomnia	1 (0.8)	Lower limit (2.5–3 mEq/L)	57 (47)
Tinnitus	1 (0.8)	Low (<2.5 mEq/L)	41 (34%)
Perspiration	1 (0.8)	Calcium	
Constipation	1 (0.8)	Normal range (2.2–2.7 mmol/L)	42 (34)
*Complications*		Low	13 (11)
Metabolic alkalosis	7 (5.7)	High	2 (1.6)
Hypokalemic paralysis	6 (4.9)	*Magnesium*	
Hypokalemia	5 (4)	Normal range (0.70–1.0 mmol/L)	19 (16)
Prolonged QT intervals	5 (4)	Low	69 (57)
Pseudogout	4 (3.3)	High	6 (5)
Rhabdomyolysis	4 (3.3)	*Urine analysis*	
ST depression	2 (1.6)	*Sodium*	
T wave changes on EKG	2 (1.6)	mmol/24h	
Gestational diabetes mellitus	2 (1.6)	Normal (40–220 mmol/24 h)	17 (14)
Focal segmental glomerulosclerosis	2 (1.6)	High	14 (11)
Prominent U waves	2 (1.6)	Spot (mmol/L)	
Tubulointerstitial nephritis	2 (1.6)	Normal (<20 mmol/L)	1 (0.8)
Brain calcification	1 (0.8)	High	3 (2.5)
Diabetic ketoacidosis	1 (0.8)	*Potassium*	
Left ventricular dysfunction	1 (0.8)	mmol/24h	
Prolonged PR interval	1 (0.8)	Normal (25–125 mmol/24 h)	30 (25)
Ventricular fibrillation	1 (0.8)	High	8 (6.5)
MPGN	1 (0.8)	Low	2 (1.6)
Focal seizures	1 (0.8)	Spot (mmol/L)	
Iron deficiency anemia	1 (0.8)	Normal (20–40 mmol/L)	3 (2.5)
Pericardial effusion	1 (0.8)	High	3 (2.5)
Neuropsychological symptoms	1 (0.8)	*Calcium*	
Sclerochoroidal calcifications	1 (0.8)	mmol/24h	
Renal tubular acidosis	1 (0.8)	Normal (15–20 mmol/24 h)	2 (1.6)
		High	3 (2.5)
*Diagnosis*		Low	58 (48)
Based on electrolyte abnormality	68 (56)	Spot (mmol/L)	
Genetic mutations		Normal (20–40 mmol/L)	
SLC12A3 gene mutations	46 (38)	High	
NCCT gene	3 (2.5)	*Magnesium*	
TSC gene	3 (2.5)	mmol/24h	
Screening	2 (1.6)	Normal (3–5 mmol/24 h)	5 (4)
CLCNKB gene	1 (0.8)	High	13 (11)
		Low	7 (6)
*Management*		Spot (mmol/L)	
Electrolyte replacement (Mg, K supplements)	92 (75)	Normal (8–152 mmol/L)	1 (0.8)
Spironolactone	32 (26)	High	N/A
Pain killers	13 (11)	Low	1 (0.8)
Angiotensin receptor blocker	7 (5.7)	*Chloride (140–250 mmol/24 h)*	
Amiloride	7 (5.7)	mmol/24 h	
Steroids	5 (4)	Normal(140–250 mmol/24 h)	2 (1.6)
Eplerenone	3 (2.5)	High	3 (2.5)
Colchicine (for gout)	2 (1.6)	Low	3 (2.5)
Desmopressin	2 (1.6)	mmol/L	
Growth hormone (for empty sella syndrome)	2 (1.6)	Normal (98–107 mmol/L)	
Febuxostat (for gout)	1 (0.8)	Low	6 (4.9)
Cyclophosphamide	1 (0.8)	High	4 (3.3)
Triamterene	1 (0.8)	*24 hr urinary protein*	
Phenytoin	1 (0.8)	Normal (<80 mg/24 h)	1 (0.8)
Amiodarone (for ventricular fibrillation)	1 (0.8)	Low	
Metoclopramide	1 (0.8)	High	7 (6)
Antithyroid drugs	1 (0.8)	*Calcium creatinine ratio*	
*Outcome*		Normal (<0.14)	
Recovery	86 (70)	High	
		Low	
